# Plasma metabolites and lipids associate with kidney function and kidney volume in hypertensive ADPKD patients early in the disease course

**DOI:** 10.1186/s12882-019-1249-6

**Published:** 2019-02-25

**Authors:** Kyoungmi Kim, Josephine F. Trott, Guimin Gao, Arlene Chapman, Robert H. Weiss

**Affiliations:** 1 Division of Biostatistics, Department of Public Health Sciences, University of California, Davis, CA USA; 2 Division of Nephrology, Department of Internal Medicine, University of California, Genome and Biomedical Sciences Building, Room 6311, 451 Health Sciences Dr, Davis, CA 95616 USA; 3 Cancer Center, University of California, Davis, CA USA; 4 Department of Public Health Sciences, University of Chicago, Chicago, IL USA; 5 Nephrology Section, University of Chicago, Chicago, IL USA; 6 Medical Service, VA Northern California Health Care System, Sacramento, CA USA

**Keywords:** ADPKD, Metabolomics, Progression, HALT study

## Abstract

**Background:**

Autosomal dominant polycystic kidney disease (ADPKD) is the most common hereditary kidney disease and is characterized by gradual cyst growth and expansion, increase in kidney volume with an ultimate decline in kidney function leading to end stage renal disease (ESRD). Given the decades long period of stable kidney function while cyst growth occurs, it is important to identify those patients who will progress to ESRD. Recent data from our and other laboratories have demonstrated that metabolic reprogramming may play a key role in cystic epithelial proliferation resulting in cyst growth in ADPKD. Height corrected total kidney volume (ht-TKV) accurately reflects cyst burden and predicts future loss of kidney function. We hypothesize that specific plasma metabolites will correlate with eGFR and ht-TKV early in ADPKD, both predictors of disease progression, potentially indicative of early physiologic derangements of renal disease severity.

**Methods:**

To investigate the predictive role of plasma metabolites on eGFR and/or ht-TKV, we used a non-targeted GC-TOF/MS-based metabolomics approach on hypertensive ADPKD patients in the early course of their disease. Patient data was obtained from the HALT-A randomized clinical trial at baseline including estimated glomerular filtration rate (eGFR) and measured ht-TKV. To identify individual metabolites whose intensities are significantly correlated with eGFR and ht-TKV, association analyses were performed using linear regression with each metabolite signal level as the primary predictor variable and baseline eGFR and ht-TKV as the continuous outcomes of interest, while adjusting for covariates. Significance was determined by Storey’s false discovery rate (FDR) q-values to correct for multiple testing.

**Results:**

Twelve metabolites significantly correlated with eGFR and two triglycerides significantly correlated with baseline ht-TKV at FDR q-value < 0.05. Specific significant metabolites, including pseudo-uridine, indole-3-lactate, uric acid, isothreonic acid, and creatinine, have been previously shown to accumulate in plasma and/or urine in both diabetic and cystic renal diseases with advanced renal insufficiency.

**Conclusions:**

This study identifies metabolic derangements in early ADPKD which may be prognostic for ADPKD disease progression.

**Clinical trial:**

HALT Progression of Polycystic Kidney Disease (HALT PKD) Study A; Clinical www.clinicaltrials.gov identifier: NCT00283686; first posted January 30, 2006, last update posted March 19, 2015.

**Electronic supplementary material:**

The online version of this article (10.1186/s12882-019-1249-6) contains supplementary material, which is available to authorized users.

## Background

Autosomal dominant polycystic kidney disease (ADPKD) is the most common hereditary kidney disorder and affects 1/1000 individuals. It is characterized by gradual enlargement of numerous cysts in the kidneys over decades, and the disease process begins long before loss of estimated glomerular filtration rate (eGFR) occurs. There are at least three definite genetic causes of ADPKD. The majority of ADPKD cases (~ 75%) are caused by mutations in polycystin 1 [1], and second most common (~ 15%) are mutations in polycystin 2 (PKD2) [2]. Recently a third causative gene in ADPKD and autosomal dominant polycystic liver disease (ADPLD) was identified to be GANAB, responsible for 0.3% of all ADPKD [3]. Mutations in GANAB result in a defect in the maturation of PKD1 such that it fails to localize in the plasma membrane [3]. PKD1 binds to PKD2 [4] and this protein complex signals tubular morphogenesis through the formation of an ion-channel [5]. When GANAB is mutated and PKD1 maturation is mostly blocked, then PKD1 doesn’t interact correctly with PKD2 and PKD2 fails to localize in the cilia [3]. This leaves 5–10% of ADPKD patients with no detectable mutation after DNA sequencing of their PKD1 and PKD2 genes [6].

The course of ADPKD is variable depending not only on which gene is mutated [7] and the strength of the mutation [8], but also on environmental factors such as dietary sodium intake [7] and smoking exposure [9], both of which contribute to disease severity and progression. A high sodium intake could contribute to an increased rate of disease progression in ADPKD via various possible mechanisms [10] and not just by its effects on hypertension. Smoking has been found to be associated with established proteinuria and this is highly associated with hypertension and a more severe renal cystic phenotype [9]. Hypertension is common and occurs early in ADPKD, prior to loss of kidney function, and is associated with more rapid disease progression and increased kidney size [11, 12]. With the recognition that ADPKD is at least in part a metabolic disease as has become evident with the discovery that glucose, histidine, glutamine, and arginine metabolic pathways are reprogrammed [13–16], there is the opportunity to investigate such pathways to develop new therapies for this disease.

With the continuing advent of new therapeutic approaches, identifying patients at risk for progression to ESRD has become paramount. The Consortium for Radiologic Imaging Studies of Polycystic Kidney Disease (CRISP) study established ht-TKV as assessed by magnetic resonance imaging as an accurate and reliable measure of renal cyst burden, which also strongly predicts those patients who will progress to advanced chronic kidney disease (CKD) [17]. Ht-TKV is now approved by the FDA as both a prognostic and a clinical trial enrichment imaging biomarker for ADPKD [18]. We asked whether plasma metabolomic analyses can further identify circulatory signatures which are indicative of increased ht-TKV or kidney function (eGFR) in individuals at risk for progression to ESRD early in the course of disease. Such studies will shed light on mechanisms of disease, including metabolic reprogramming. The HALT A clinical trial was designed and developed to test the hypothesis that rigorous blood pressure control (< 110/75 mmHg) or combined angiotensin converting enzyme inhibitor and angiotensin receptor blocking therapy would slow disease progression defined as the rate of increase in ht-TKV and the chronic slope of decline in eGFR. Importantly, low blood pressure control was found to have a significant protective impact on both the rate of ht-TKV increase as well as the chronic eGFR slope. For this study, we now have utilized plasma from the baseline visit from participants in the HALT-A randomized clinical trial [12, 19], after stopping all antihypertensive medications for at least two weeks, in an attempt to correlate expression of specific metabolites with simultaneously measured eGFR and ht-TKV.

## Methods

### Ethics approval and consent to participate

IRBs at each participating center approved the HALT-A clinical trial. These included University of Colorado Health Sciences Center, Denver (Aurora), Colorado; Emory University School of Medicine, Atlanta, Georgia; University of Kansas Medical Center, Kansas City, Kansas; Tufts Medical Center, Boston, Massachusetts; Beth Israel Deaconess, Boston, Massachusetts; Cleveland Clinic, Cleveland, Ohio; Mayo Clinic, Rochester, Minnesota. All subjects provided written consent, and parental consent was required and obtained for all participating subjects between the ages of 15 and 18 years of age at each participating site. Two Data Coordinating Centers were involved in the HALT-A clinical trial: Washington University, St. Louis Missouri and the University of Pittsburgh, Pittsburgh, Pennsylvania. IRB study approval was obtained at each Data Coordinating Center.

### Subject enrollment

This study was approved by the Institutional Review Boards both at the University of California, Davis, and at the University of Chicago. The HALT Progression of Polycystic Kidney Disease (HALT PKD, NCT00283686, http:// clinicaltrials.gov) Study A is a double-blind, placebo-controlled trial consisting of 588 hypertensive participants (15 to 49 years of age, with eGFR > 60 ml/1.73 m^2^) [12]. Briefly, subjects provided written consent and were enrolled, after which antihypertensive medications were withdrawn for two weeks. Patients who demonstrated elevations in blood pressure during washout were placed on labetolol therapy. At baseline, blood was obtained for centralized eGFR measures, MR imaging performed for ht-TKV measurement and plasma and urine collected and appropriately stored for future analyses. Plasma samples were handled immediately at each participating clinical center and stored within 30 min at − 80 degrees. Samples were shipped in bulk to the NIDDK repository for storage prior to this study. Subjects were randomized in a 2 × 2 factorial design to determine whether rigorous blood pressure control (< 110/75 mm HG vs. < 130/80 mmHg) or additive inhibition of the renin-angiotensin-aldosterone-system (RAAS) impacted disease progression. The primary outcomes of our study were baseline ht-TKV and eGFR measures. We analyzed baseline fasting plasma samples obtained from a subset of 277 Caucasian ADPKD patients who were known to have mutations in either the PKD1 and PKD2 genes.

### Non-targeted metabolomics analysis

Samples and standards were analyzed as described [20]. Peaks were deconvoluted and detected using Leco ChromaTOF software and were matched to FiehnLib mass spectra and retention time libraries. The Binbase software was used for post-curation and peak replacements, and the sum of intensities for all known compounds was used to normalize data.

### Non-targeted lipidomics analysis

Samples and standards were analyzed as described [21]. The sample temperature was maintained at 4 °C.

### Statistical analysis

Metabolomic and lipidomic analyses were performed on all 277 plasma samples by gas chromatography-time of flight mass spectrometry (GC-TOF/MS). Patient samples were analyzed along with 35 (metabolomics) and 36 (lipidomics) splits of the “reference” (pooled) quality control sample placed evenly in between patient samples for quality control assessment. Run order of patient samples was randomly determined. Note that for the lipidomics data, one patient sample showed anomalous behavior and was markedly dispersed from the rest of the patient samples (i.e., contained values above and below 3 standard deviations from the mean) and thus removed from further analysis.

A total of 116 plasma metabolites and 101 lipids were identified, quantitated, and included in downstream statistical analysis. Also 174 unknown plasma metabolites were analyzed separately in the same manner described below. For lipidomic data, lipids quantified in fewer than 50% of samples were discarded from downstream analysis to reduce the bias that could be induced by imputation due to the detection limit. Unobserved values for any remaining undetected lipid below the pre-defined detection limit were imputed as one-half of the lipid-specific minimum of the observed values prior to normalization and statistical inference tests.

For between-sample normalization, the intensity values for each sample were summed, and the median value of the sums across all samples were determined. The intensity values of each sample were then scaled such that the sum of the scaled intensities equaled the median value of all samples. Thus, the sum of the scaled intensities was the same for all samples. Normalized intensity values were then log_2_ transformed to reduce the influence of extreme values and to meet the homogeneity of variance assumption.

Prior to assessing the association between metabolite intensity levels and disease severity (baseline eGFR and ht-TKV), we assessed the distributions of eGFR and ht-TKV and then log transformed them to meet normality and homoscedasticity of linear regression models. To identify individual metabolites whose intensities are significantly associated with eGFR and ht-TKV, we performed association analyses using linear regression with each metabolite signal level as the primary predictor variable and baseline eGFR and ht-TKV as the two outcomes of interest, analyzed separately. All analyses accounted for the covariates attributed to the HALT-A study design including age, sex, PKD genotype (PKD1 or PKD2), and participating clinical site of sample collection. Other HALT covariates, including baseline systolic and diastolic blood pressure, weight, body mass index (BMI), height-adjusted liver volume, and urine levels for sodium, potassium, and creatinine, were evaluated for inclusion in multiple linear regression models to determine whether any of those covariates are influential in estimating the magnitude of association of metabolite levels with the outcomes. Variable inclusion and exclusion were determined based on Akaike weight-based relative importance (RI) scores [22], with a suggestive threshold of 0.80 in conjunction with likelihood ratio tests (LRT) at *p*-value < 0.05. Multicollinearity was inspected for possible collinearity issues using variance inflation factor (VIF) score with a suggestive threshold of 2.5. We investigated potential effect modifiers such as sex and PKD genotype by adding interaction terms in regression models. When an interaction term reached statistical significance, post-hoc analysis was performed stratified by the effect modifier. Five missing values for BMI were imputed with the mean of the observed values. We used the Storey’s false discovery rate (FDR) q-values to correct for multiple testing [23]. All statistical analyses were conducted in R (version 3.4.) language and environment.

## Results

### Reproducibility of metabolomics and lipidomics data

Due to the large number of samples (277; Table 1) and the necessity to process the samples over a prolonged time period of several days, we first investigated reproducibility and time-dependent variation using the repeated measurements from the 35 and 36 technical replicates, for metabolomics and lipidomics respectively of the reference quality control sample which were inserted throughout the GC-TOF/MS analysis to assess for systematic shifts among samples. Boxplots of the intensity values of the reference sample replicates revealed no systematic time-dependent pattern in the magnitude or distribution of intensities (Additional file 1: Figure S1 and S2). In order to provide a direct quantitative measure of technical between-sample variability, we calculated the coefficient of variation (CV) across the 35 and 36 measurements for each metabolite and lipid in the reference sample. The CV values of the 35–36 replicates ranged from 0.006 to 0.445 with mean = 0.051 and SD = 0.045 for metabolites and from 0.232 to 0.250 with mean = 0.241 and SD =0.004 for lipids. These low CV values demonstrate adequate technical reproducibility of sample analyses during the course of the GC-TOF/MS experiment.

**Table 1:** Summary statistics of patient characteristics with metabolomics data

Variable	Caucasian Patients (*n* = 277)	
	Mean (SD)	N of Missing
Age (years)	36.87 (8.11)	0
Sex		0
Female, no (%)	132 (47.7%)	
Male, no (%)	145 (52.3%)	
PKD Genotype		0
PKD1, no (%)	226 (81.6%)	
PKD2, no (%)	51 (18.4%)	
Body Mass Index (BMI)	27.03 (5.05)	5
Height-Adjusted Liver Volume, mL	1147.93 (525.12)	9
Diastolic Blood Pressure, mmHg	80.02 (10.37)	2
Systolic Blood Pressure, mmHg	126.23 (12.85)	2
Urine Creatinine Level (mEq/day)	1478.97 (742.86)	3
Urine Potassium Level (mEq/day)	56.41 (28.17)	7
Urine Sodium Level (mEq/day)	174.82 (85.20)	3
Height-Adjusted Total Kidney Volume (ht-TKV, mL)	721.62 (420.75)	4
Estimated glomerular filtration rate (eGFR, ml/min)	90.55 (18.19)	0

### Selection of accountable covariates in association with metabolite signals of baseline eGFR and ht-TKV

**Fig. 1:**
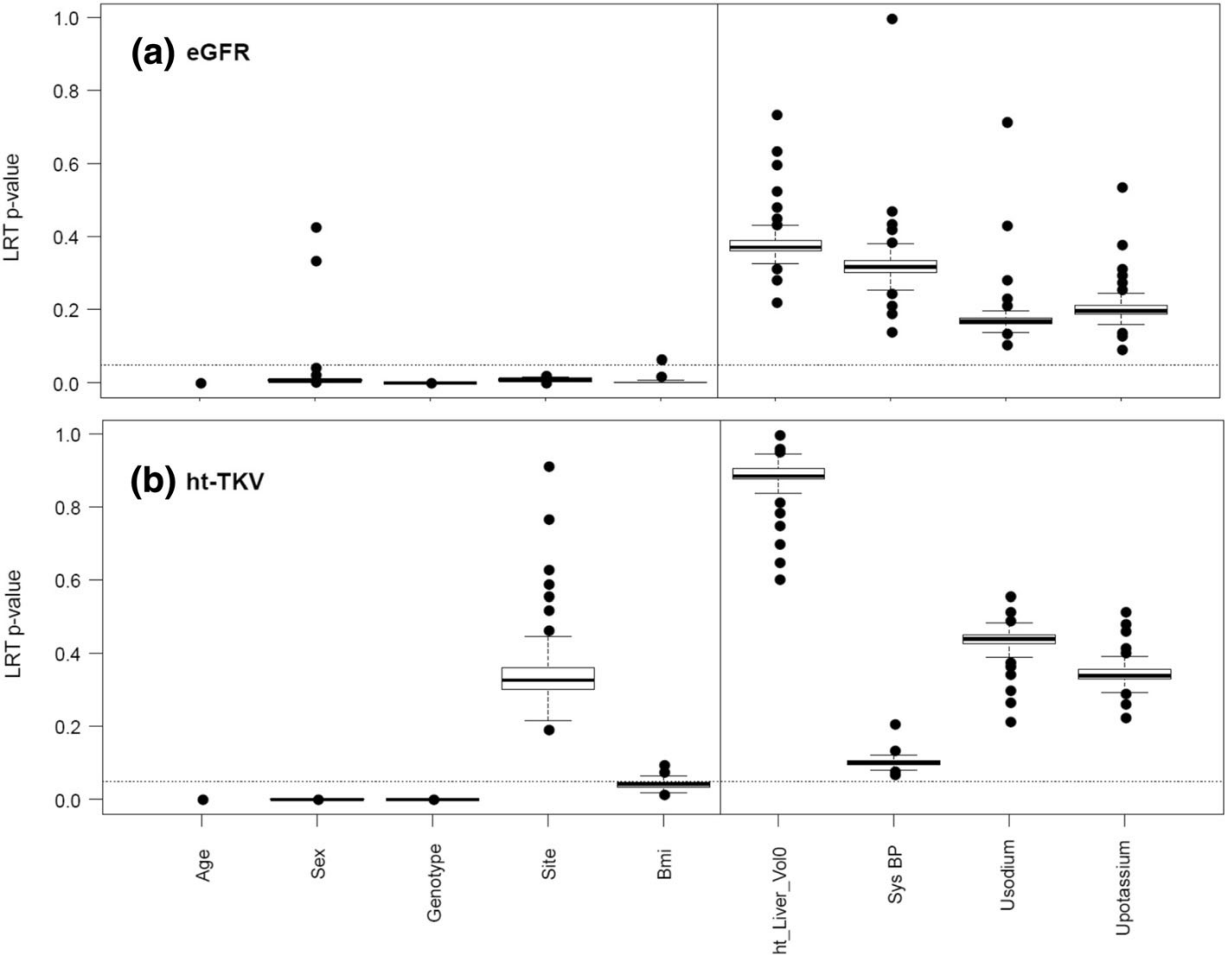
Boxplots of likelihood ratio test (LRT) *p*-values of 290 metabolites determining significance of each of the HALT covariates to include in the final analysis models for both (**a**) eGFR and (**b**) ht-TKV. The dashed line represents the *p* = 0.05 significance level

To determine which covariates should be included in multiple regression models, Akaike weight-based RI scores were calculated as a merit for importance of inclusion of each covariate in a model for all metabolites (ranged from 0.29 to 0.905 for eGFR and from 0.28–0.812 for ht-TKV) and final variable selection was carried out via likelihood ratio test (LRT). Age, genotype, sex, site of sample collection, and BMI were significantly (*p* < 0.05) influential in association between metabolites and outcomes, eGFR and ht-TKV (Fig. 1) and thus we retained these covariates in the final models. Although the site of sample collection was not significant for ht-TKV at *p*-value < 0.05, we decided to include it in the final model to account for possible study site differences. The variable selection procedures led to the following final models for association analysis explicitly:

1. eGFR = α + β_1_*M_Signal + β_2_*Age + β_3_*Genotype + β_4_*Sex + β_5_*Site + β_6_*BMI.
2. ht-TKV = α + β_1_*M_Signal+ β_2_*Age + β_3_*Genotype + β_4_*Sex + β_5_*Site + β_6_*BMI.

where M_ Signal is the signal level of a given metabolite/lipid.

We further investigated potential multicollinearity issues among the selected variables in the above models and found no presence of collinearity.

### Identification of plasma metabolites and lipids associated with baseline eGFR and ht-TKV

**Fig. 2:**
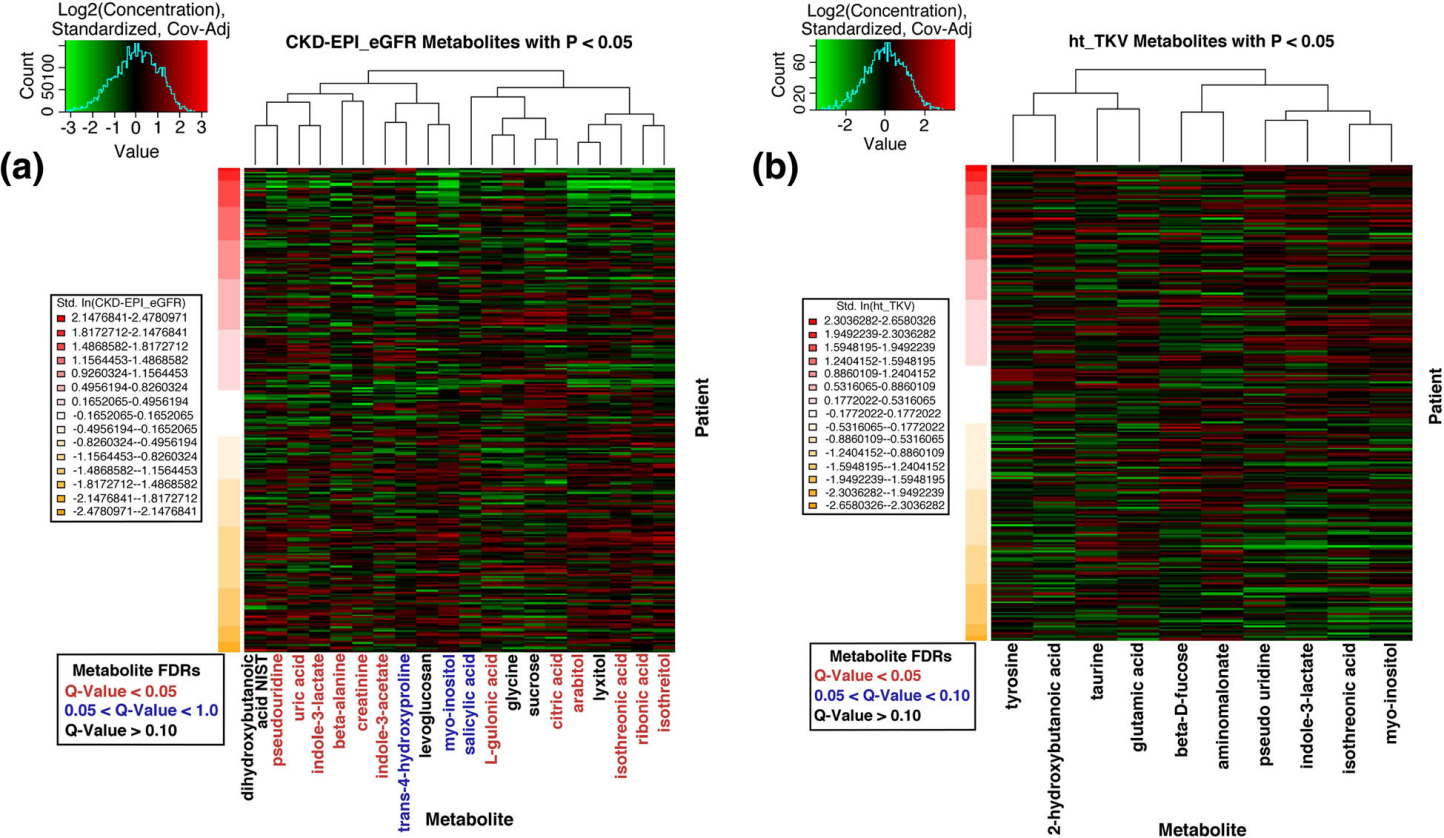
Heatmap of significantly (p < 0.05) associated metabolites with (**a**) eGFR and (**b**) ht-TKV. Rows: patients sorted by their outcome values from high to low; Columns: metabolites. Dendrogram clustering on the X-axis indicates metabolite similarity. Expression values are log_2_ transformed. Red and green color intensity indicate positive and negative intensity

After accounting for the covariates discussed above, we discovered that 20 metabolites significantly associated with eGFR at *p* < 0.05. Of those, 12 metabolites remained significant at FDR q-value < 0.05 (Table 2). All of the significant metabolites exhibited negative associations with eGFR, meaning that the metabolite levels were increased with decreasing eGFR levels (Fig. 2). Of note, indole-3-lactate is an exogenously produced microbial metabolite, while pseudo-uridine is a known uremic toxin (see Discussion).

**Table 2:** Plasma metabolites significantly (*p* < 0.05) associated with baseline eGFR. Effect size represents the magnitude and direction (+/−) of association between a given metabolite and eGFR, estimated by linear regression, while controlling for the selected HALT covariates, age, sex, genotype, site of sample collection, and BMI. KEGG ID is the metabolite identifier for Kyoto Encyclopedia of Genes and Genomes (KEGG) and PubChem ID is for the chemical molecule database maintained by the National Center for Biotechnology Information

Metabolite	Effect Size (SD)	*p*-value	FDR q-value	KEGG ID	PubChem ID
pseudo uridine	−0.1796 (0.023)	< 0.0001	***< 0.0001***	C02067	15,047
indole-3-lactate	−0.1204 (0.021)	< 0.0001	***< 0.0001***	C02043	92,904
creatinine	−0.0616 (0.016)	0.0002	***0.0040***	C00791	588
isothreonic acid	−0.0616 (0.017)	0.0004	***0.0076***	C21649	151,152
uric acid	−0.0508 (0.016)	0.0016	***0.0249***	C00366	1175
beta-alanine	−0.0369 (0.012)	0.0027	***0.0349***	C00099	239
isothreitol	−0.0504 (0.017)	0.0034	***0.0369***	C16884	169,019
Arabitol	−0.0431 (0.015)	0.0038	***0.0369***	C01904	94,154
ribonic acid	−0.0577 (0.020)	0.0049	***0.0410***	C01685	5,460,677
indole-3-acetate	−0.0450 (0.016)	0.0061	***0.0410***	C00954	802
citric acid	−0.0685 (0.025)	0.0063	***0.0410***	C00158	311
L-gulonic acid	−0.0605 (0.022)	0.0063	***0.0410***	C00800	6,857,417
salicylic acid	−0.0166 (0.006)	0.0092	0.0532	C00805	338
myo-inositol	−0.0421 (0.016)	0.0096	0.0532	C00137	892
trans-4-hydroxyproline	−0.0428 (0.017)	0.0115	0.0595	C01157	5810
Sucrose	−0.0210 (0.009)	0.0237	0.1148	C00089	5988
2,3-dihydroxybutanoic acid NIST	−0.0364 (0.017)	0.0294	0.1269		250,402
Glycine	−0.0492 (0.022)	0.0295	0.1269	C00037	750
levoglucosan	−0.0176 (0.008)	0.0342	0.1396		2,724,705
Lyxitol	−0.0244 (0.012)	0.0463	0.1796	C00532	439,255

bold Q-values indicate signficance, <0.05

We next evaluated metabolites that associated with ht-TKV. Ten metabolites were found to be significantly associated with ht-TKV at *p* < 0.05; however, none of them maintained their significance at FDR q-value < 0.05 (Table 3). Four metabolites (taurine, tyrosine, glutamic acid, and 2-hydoxybutanoic acid) were negatively correlated with ht-TKV, while the others exhibited a positive correlation (Fig. 2).

**Table 3:** Plasma metabolites significantly (*p* < 0.05) associated with baseline ht-TKV. Effect size represents the magnitude and direction (+/−) of association between a given metabolite and ht-TKV, estimated by linear regression, while controlling for the selected HALT covariates, age, sex, genotype, site of sample collection, and BMI

Metabolite	Effect Size (SD)	*p*-value	FDR q-value	KEGG ID	PubChem ID
pseudo uridine	0.2273 (0.082)	0.0061	0.3375	C02067	15,047
aminomalonate	0.1240 (0.047)	0.0094	0.3375	C00872	100,714
Taurine	−0.0577 (0.024)	0.0151	0.3375	C00245	1123
Tyrosine	−0.1984 (0.082)	0.0156	0.3375	C00082	6057
isothreonic acid	0.1333 (0.055)	0.0166	0.3375	C21649	151,152
glutamic acid	−0.1111 (0.048)	0.0217	0.3677	C00025	33,032
indole-3-lactate	0.1544 (0.070)	0.0273	0.3965	C02043	92,904
myo-inositol	0.1080 (0.052)	0.0373	0.4075	C00137	892
2-hydroxybutanoic acid	−0.0940 (0.045)	0.0384	0.4075	C05984	440,864
Beta-D-fucose	0.0913 (0.046)	0.0482	0.4075	C02095	439,650

In analyzing the lipidome, we discovered 11 lipids which were significantly associated with eGFR at *p* < 0.05. However, no lipids remained significant at FDR q-value < 0.05. Acylcarnitines and triglycerides exhibited negative associations with eGFR, and phosphatidylcholines and diglyceride exhibited positive associations (Table 4). Upon analysis of the relationship between the lipidome and ht-TKV, we found that five triglycerides and one sphingomyelin were significantly associated with ht-TKV at *p* < 0.05, however, of these only two triglycerides maintained their significance at FDR q-value < 0.05 (Table 5). All six of these lipids exhibited positive associations with ht-TKV.

**Table 4:** Plasma lipids significantly (*p* < 0.05) associated with baseline eGFR. Effect size represents the magnitude and direction (+/−) of association between a given lipid and eGFR, estimated by linear regression, while controlling for the selected HALT covariates, age, sex, genotype, site of sample collection, and BMI

Lipid	Effect Size (SD)	*p*-value	FDR q-value
Acylcarnitine(10:1) [M + H]+	−0.0424 (0.013)	0.0010	0.0737
Triglyceride(53:3) [M + K]+	−0.0516 (0.017)	0.0033	0.1024
Acylcarnitine(14:2) [M + H]+	−0.0303 (0.011)	0.0057	0.1024
Acylcarnitine(12:1) [M + H]+	−0.0350 (0.013)	0.0059	0.1024
Acylcarnitine(8:0) [M + H]+	−0.0291 (0.011)	0.0071	0.1024
Triglyceride(54:5) A [M + K]+	−0.0470 (0.020)	0.0199	0.2364
Phosphatidylcholine(28:0) [M + H]+	0.0275 (0.012)	0.0229	0.2364
Phosphatidylcholine(38:7) [M + H]+	0.0578 (0.028)	0.0397	0.2959
Triglyceride(59:3) [M + NH4]+	−0.0320 (0.015)	0.0399	0.2959
Diglyceride(32:0) [M + K]+	0.0506 (0.025)	0.0409	0.2959
Phosphatidylcholine(30:0) [M + H]+	0.0307 (0.015)	0.0477	0.3137

**Table 5:** Plasma lipids significantly (*p* < 0.05) associated with baseline ht-TKV. Effect size represents the magnitude and direction (+/−) of association between a given lipid and ht-TKV, estimated by linear regression, while controlling for the selected HALT covariates, age, sex, genotype, site of sample collection, and BMI

Lipid	Effect Size (SD)	*p*-value	FDR q-value
Triglyceride(51:3) [M + K]+	0.1626 (0.038)	< 0.0001	***0.0031***
Triglyceride(53:3) [M + K]+	0.1827 (0.055)	0.0010	***0.0492***
Sphingomyelin(d43:1) [M + H]+	0.1560 (0.070)	0.0276	0.5728
Triglyceride d5(17:0/17:1/17:0) iSTD [M + Na]+	0.1038 (0.049)	0.0337	0.5728
Triglyceride(54:5) A [M + K]+	0.1350 (0.064)	0.0352	0.5728
Triglyceride(52:3) [M + K]+	0.1719 (0.082)	0.0360	0.5728

bold Q-values indicate signficance, <0.05

### Assessment of effect modification by sex or PKD genotype

**Fig. 3:**
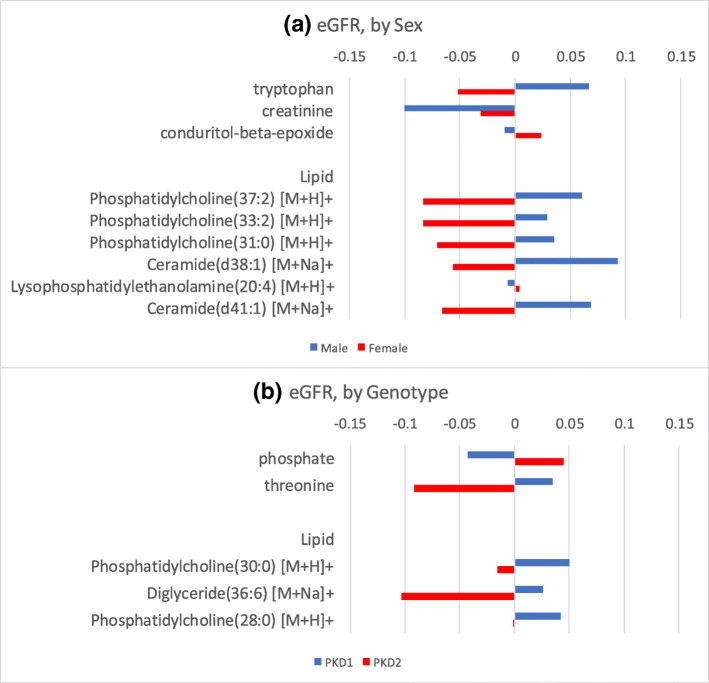
Altered associations between a given metabolite/lipid and outcome, eGFR, by effect modifiers sex and genotype. A different color represents a different sex and genotype. The length and direction of bar indicate a degree and direction (negative or positive) of association with that metabolite/lipid respectively (see Additional file 2: Tables S1 and S2 for details)

We further investigated whether the relationship between metabolite level and eGFR or ht-TKV differ depending on patient’s sex or their PKD genotype, to evaluate the possibility that either could have a significant role in metabolomics signature expression in association with eGFR or ht-TKV. To accomplish this, we added an interaction term to the model depicted by equation 1) above for eGFR and to the model depicted by equation 2) above for ht-TKV. For eGFR, none of the metabolites showed significant effect modification by sex or genotype at FDR q-value< 0.05 (Additional file 2: Table S1). For significant metabolites at *p* < 0.05, the direction of the metabolite-eGFR association was generally reversed by sex. Positive metabolite-eGFR associations for females were altered to negative associations for males, and vice-versa; similar data were demonstrated for metabolite-PKD genotype associations (Fig. 3). By contrast, creatinine exhibited negative associations for both males and females, but at different degrees: the magnitude of association was 3-fold greater in males compared to females (Fig. 3 (a), Additional file 2: Table S1).

**Fig. 4:**
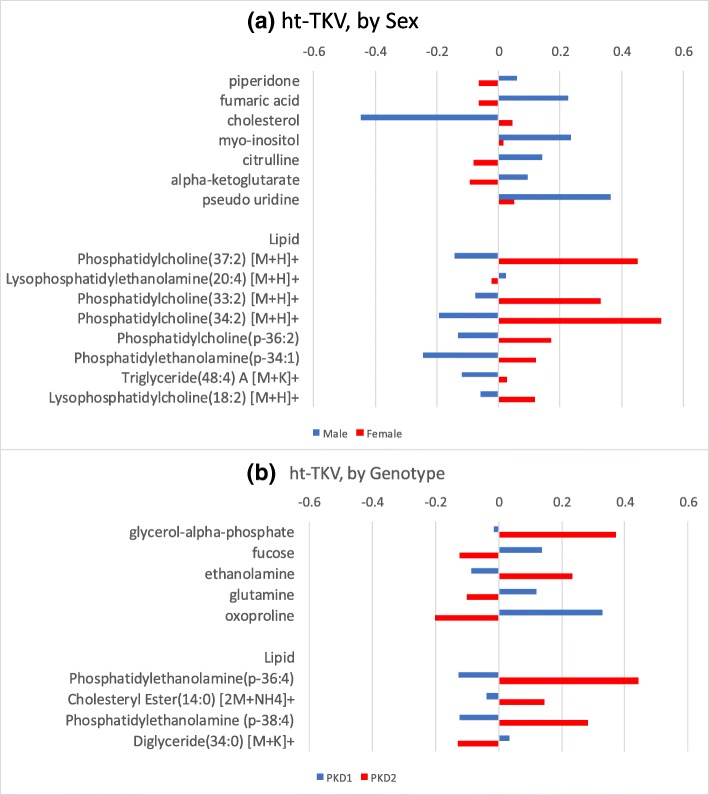
Altered associations between a given metabolite/lipid and outcome, ht-TKV, by effect modifiers sex and genotype. A different color represents a different sex and genotype. The length and direction of bar indicate a degree and direction (negative or positive) of association with that metabolite/lipid respectively (see Additional file 2: Tables S1 and S2 for details)

For ht-TKV, none of the metabolites showed significant effect modification by sex or genotype at FDR q-value < 0.05 (Additional file 2: Table S2). The majority of the metabolites that displayed significant effect modification by sex or genotype at *p* < 0.05 modified the direction of the metabolite-ht-TKV association (Fig. 3). Pseudo-uridine and myo-inositol exhibited positive association regardless of sex, but their magnitude of association differed depending on sex. The degree of association for pseudo-uridine was 7-fold greater in males compared to females, and for myo-inositol was 15-fold greater in males than females (Fig. 4(a), Additional file 2: Table S2).

For both eGFR and ht-TKV, no lipids showed significant effect modification by sex or genotype at FDR q-value < 0.05 (Additional file 1: Tables S1 & S2). The majority of those lipids significant at *p*-value < 0.05 had the directionality of their associations with eGFR (Fig. 3) and ht-TKV (Fig. 4) altered by sex and genotype, respectively. It should be noted that none of the metabolites and lipids reached FDR-adjusted significance after taking multiple testing into account and thus further investigation is needed to validate whether sex or genotype are necessary to treat them as effect modifiers in the associations with eGFR or ht-TKV for metabolites or lipids.

### Association of metabolites and lipids with eGFR and ht-TKV are exclusively independent

In the above results, we have identified metabolites and lipids associated with eGFR and ht-TKV. As eGFR and ht-TKV are clinically correlated traits for disease severity, we decided to perform further analysis using those outcomes as covariates in an attempt to identify metabolites associated only with eGFR or ht-TKV exclusively independent of each other. To accomplish this, ht-TKV was added as an additional covariate to the model depicted by equation 1) for eGFR as previously described, while eGFR was added as an additional covariate to the model depicted by equation 2) for ht-TKV. When controlled for ht-TKV as a covariate, four metabolites were determined to be significantly associated with eGFR at FDR q-value < 0.05, including pseudo-uridine, indole-3-lactate, creatinine, and uric acid (Additional file 2: Table S3); all of which were among the twelve metabolites previously identified as significant at FDR q-value< 0.05 for eGFR without controlling for ht-TKV (Table 2). Directionality of the eGFR-metabolite association remained unchanged compared to the ht-TKV unadjusted analysis results. All significant metabolites exhibited negative associations with eGFR independent of ht-TKV, with the exception of pipecolinic acid which demonstrated a positive relationship with eGFR. No lipids were determined to be significant at the FDR q-value< 0.05 level (Additional file 2: Table S3).

When evaluating ht-TKV while controlling for eGFR as a covariate, only taurine was found to be significant at *p* < 0.05, although it was not significant at FDR q-value < 0.05 (Additional file 2: Table S3). Taurine was also found to be associated with ht-TKV in the previous analysis that did not control for eGFR as a covariate (Table 3). Adjustment for eGFR did not alter the directionality of association between the metabolite and ht-TKV. In addition, triglyceride (51:3) was significant at FDR q-value< 0.05, as previously found without controlling for eGFR (Table 5), while the directionality of the association remains unchanged regardless of controlling for eGFR as a covariate in the analysis. The eGFR- and ht-TKV unique metabolites therefore, suggest that even those two highly correlated traits may be mediated, at least in part, by different metabolic pathways in kidney function.

## Discussion

Many metabolic disorders have been associated with ADPKD [24], including hypocitraturia [25, 26], hypomagnesiuria [25, 27, 28], hyperuricemia and gout [29], hypophosphataemia [30], altered arginine metabolism [15], abnormal lipid metabolism [31, 32] and nephrolithiasis [25, 27]. Recently the Warburg effect metabolic change that is characteristic of cancer has been identified in ADPKD [13].C-Glucose tracking in a kidney specific PKD1-null mouse model found that cystic kidneys had increased aerobic glycolysis, and transcriptomics of human ADPKD kidneys revealed gluconeogenesis enzymes were downregulated while some glycolytic enzymes were upregulated [33]. For this report, we asked which plasma metabolites can serve as indicative of baseline eGFR and ht-TKV in the HALT A cohort of hypertensive ADPKD patients with relatively preserved (> 60 ml/min) kidney function. We found several metabolites that significantly correlated with eGFR and two lipids that correlated with ht-TKV at FDR q-value< 0.05. All of the metabolites which correlated with eGFR had plasma concentrations inversely correlated with the patient’s eGFR. Since all of the significant (FDR q < 0.05) metabolites showed negative associations with eGFR, it is possible that their increased presence is due merely to decreased filtration or tubular reabsorption. However, in light of previous findings of these metabolites in uremic serum, it is possible that disease specific changes are related to the utilization or transport of these metabolites. It is of great interest that these metabolites were seen to be increased in individuals with relatively preserved kidney function (Table 1), which suggests that there may be an early disruption of their transport or utilization in patients with kidney disease considered to be early with normal kidney function.

Non-targeted metabolomics is a powerful tool to generate hypotheses and discover novel small molecular biomarkers that may have therapeutic impact. However, there are limitations to this technique [34], some of which are apparent in this study. In most GC/MS-based metabolomics studies, it is common to come across unidentified metabolites for which a mass/charge ratio is present but structural identification is lacking. Many of these compounds can be identified by nuclear magnetic resonance or other advanced target-specific techniques, but such analysis requires prior knowledge of specific targets as well as a substantial investment and considerably larger quantities of starting material [35]. In this study, one such metabolite, 191801, was found to be highly correlated with eGFR (FDR q < 0.0001) even after controlling for ht-TKV as a covariate (Additional file 2: Table S3). While we can infer properties and the possible origin of this metabolite based on other studies in the analyzing laboratory (i.e. that it is likely of microbial origin), structural information on this metabolite at this point is lacking.

We are the first to examine the metabolome of plasma from ADPKD patients with intact kidney function. Other ADPKD metabolomics studies have been done on mouse urine [36, 37], mouse kidneys [32] and mouse cell culture media [33]. Of the twelve identified plasma metabolites which we found to be associated with eGFR, pseudo uridine and uric acid have long been known to be present in uremic serum [38] and these compounds have more recently been shown to be markers of high risk or progression to ESRD in type 2 diabetes [39]. Higher baseline serum uric acid in ADPKD patients has also been retrospectively associated with a high risk of ESRD [40] and uric acid is increased in the urine of a juvenile cystic mouse model [37]. One study found an association between increased serum uric acid and decreased creatinine clearance rate in ADPKD, without any evidence of increased uric acid production [41], a second study found ADPKD patients had a higher incidence of hyperuricemia than patients with CKD or normal renal function despite all groups having normal rates of uric acid excretion [29] and a third study found ADPKD patients with normal renal function had elevated serum uric acid [42]. Neither study found evidence of increased uric acid synthesis. The CRISP study found that higher baseline serum uric acid was not associated with declines in eGFR or increases in ht-TKV in ADPKD patients over a 6-year period [10] so the mechanism behind a higher incidence of hyperuricemia in ADPKD remains unresolved. We are currently undergoing analyses to determine if any of these 12 metabolites at baseline in ADPKD are predictive of faster disease progression. In addition, urine metabolites are being evaluated for a future study, as it is clear that there is high biomarker potential in this biofluid [8, 37, 43, 44].

Several metabolites, including indole-3-lactate and likely the unknown compound 191801, are microbial metabolites, the former being the end-product of tryptophan metabolism produced by gut microbes [45], and the latter is found in wine, microalgae, fungi and yeast, as well as in human urine and plasma and is therefore likely a microbial metabolite (please see https://binvestigate.fiehnlab.ucdavis.edu/#/bin/191801 for further analytical information on the metabolite 191801 as well as in what species it has been observed). Indole-3-lactate and indole-3-acetate are both present in the urine of a juvenile cystic mouse model [37]. Meanwhile creatinine, which is a component of the CKD-EPI eGFR equation, was found to be significantly associated with eGFR levels, which serves as a supportive positive internal control for the analysis presented here.

We found significant associations between two large chain length triglycerides and baseline values of ht-TKV in ADPKD patients. This kind of association between specific triglycerides and a marker of progression risk has not been previously described in early ADPKD patients with relatively intact kidney function. Lipid dysregulation is associated with ADPKD in mouse models, including impaired fatty acid oxidation in the kidney [32] and upregulation of apolipoprotein-related genes in PKD-1 null kidneys [31]. Fatty acid metabolism was found to be altered in ADPKD patients both with normal renal function (HALT A) and decreased renal function (HALT B) [46]. A comparison between ADPKD with normal kidney function and healthy patients found only 3 components of metabolic syndrome were increased in ADPKD patients, namely hypertension, fasting glycemia and abdominal obesity [47]. A different study of young ADPKD patients with normal renal function found increased insulin resistance, inflammation and low density lipoprotein (LDL) in ADPKD [42]. A study in Southern India found ADPKD patients of a wide variety of ages had moderately increased plasma triglycerides, LDL and very low density lipoprotein (VLDL), and a ~ 50% reduction in high density lipoprotein (HDL) compared to healthy controls [48]. The Consortium for Radiologic Imaging Studies of Polycystic Kidney Disease (CRISP) cohort 6-year study found baseline serum HDL-cholesterol levels had a protective effect against increased kidney growth and faster declines in GFR [10]. Hypertriglyceridemia is not a feature of ADPKD in contrast to CKD (reviewed in [49]), which is also characterized by changes in acylcarnitine levels (reviewed in [50]). We are currently undergoing analyses to determine if the plasma triglyceride concentrations at baseline in ADPKD are predictive of faster disease progression.

We also evaluated the metabolites when accounting for sex and genotype as possible effect modifiers in their association with outcome. We found evidence that metabolite-outcome associations might be altered by sex or by genotype. However, none of the metabolites were found to be significant at FDR q < 0.05 when controlling for multiple testing so neither sex nor PKD genotype are likely a differentiating factor in the association of all or most metabolites with those outcomes, but nonetheless further investigation is warranted.

In summary, we have shown the presence of metabolites and lipids which highly correlate with eGFR and ht-TKV in ADPKD patients with normal kidney function. These metabolites and lipids can be useful for uncovering metabolic derangements, as well as reprogramming, which appear to occur early in disease. With further evaluation we may find additional predictors of early kidney dysfunction that are potentially less costly than measuring ht-TKV, which is currently the only measurement known to predict the rate of disease progression. Perhaps these metabolites will allow for prognostication and stratification of patients into slow and rapid progressors to ESRD in ADPKD.

## Conclusions

This study identifies metabolic derangements in early ADPKD which may be prognostic for ADPKD disease progression. Such data will be useful for future studies designed to predict outcome of disease based on such early metabolic changes and will likely lead to new therapeutic paradigms for a disease with quite limited therapeutic options.

## Additional files

Additional file 1: **Figure S1.** Boxplots of the distribution of Metabolite intensity levels (on log 2 scale) for all 35 reference samples of metabolomics data. **Figure S2.** Boxplots of the distribution of Lipid intensity levels (on log 2 scale) for all 36 reference samples of lipidomics data. (PPTX 103 kb)

Additional file 2: **Table S1.** Significant (*p* < 0.05) effect modification of patient’s sex/genotype on metabolite/lipid-eGFR association. **Table S2.** Significant (*p* < 0.05) effect modification of patient’s sex/genotype on metabolite/lipid-ht-TKV association. **Table S3.** Plasma metabolites and lipids significantly (*p* < 0.05) associated with (a) eGFR, after controlling for ht-TKV as a covariate and with (b) ht-TKV, after controlling for eGFR as a covariate. (XLSX 19 kb)

## Abbreviations

ADPKD: Autosomal dominant polycystic kidney disease
BMI: Body mass index
CKD: Chronic kidney disease
CRISP: Consortium for Radiologic Imaging Studies of Polycystic Kidney Disease
CUDA: N-Cyclohexyl-N′-dodecanoic acid urea
CV: Coefficient of variation
eGFR: Estimated glomerular filtration rate
ESRD: End stage renal disease
FDR: Storey’s false discovery rate
GC-TOF/MS: Gas chromatography-time of flight/mass spectrometry
HDL: High density lipoprotein
ht-TKV: Height corrected total kidney volume
LDL: Low density lipoprotein
LRT: Likelihood ratio tests
MTBE: Methyl *tert*-butyl ether
RAAS: Renin-angiotensin-aldosterone-system
RI: Relative importance
VIF: Variance inflation factor
VLDL: Very low density lipoprotein
