# Supplementary figures and images for: Plasma metabolites and lipids associate with kidney function and kidney volume in hypertensive ADPKD patients early in the disease course
Source: BMC Nephrol. 2019 Feb 25;20:66. doi: 10.1186/s12882-019-1249-6 (PMC6388487; doi:10.1186/s12882-019-1249-6)

## Slide 1
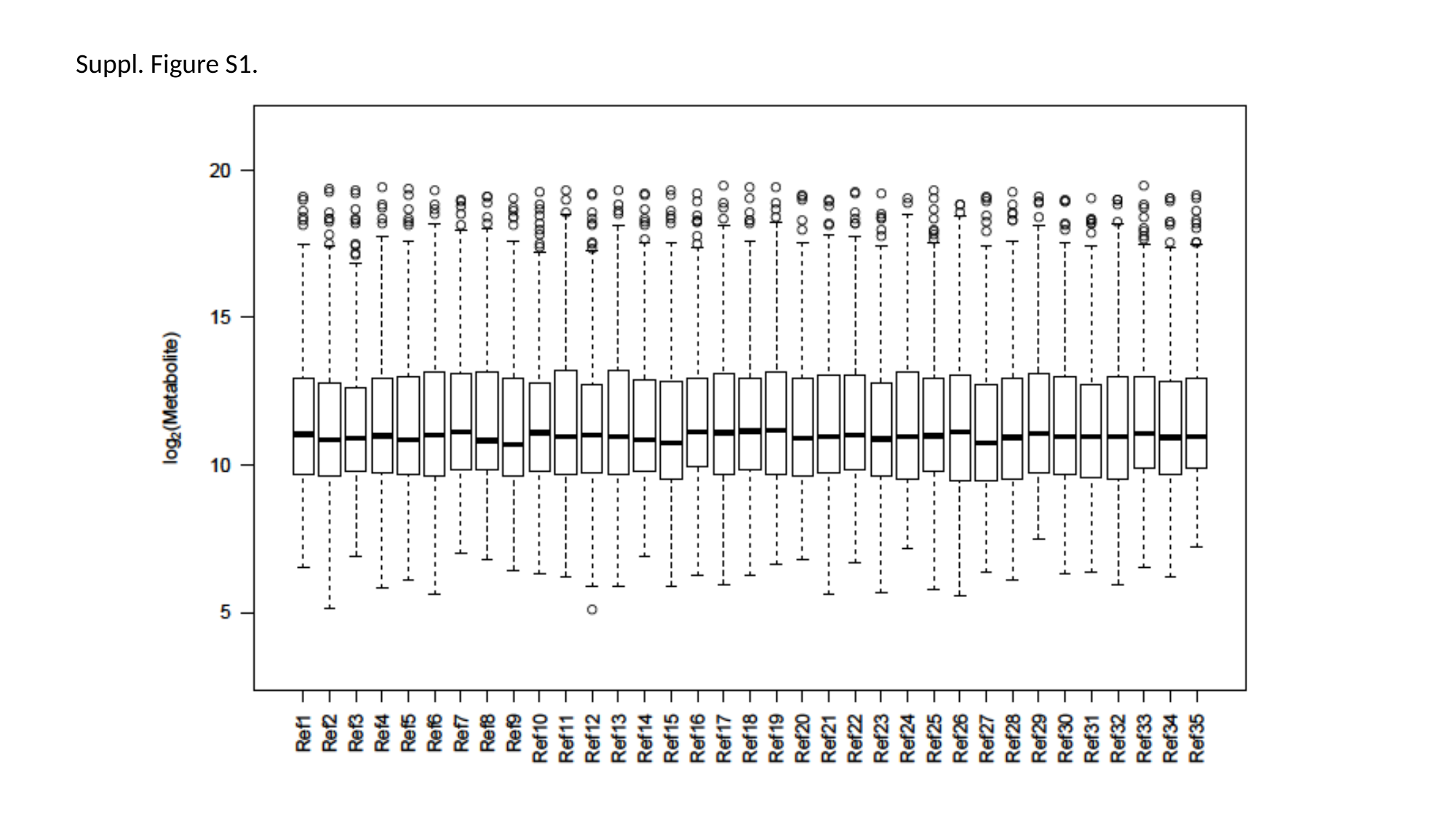

Suppl. Figure S1.

## Slide 2
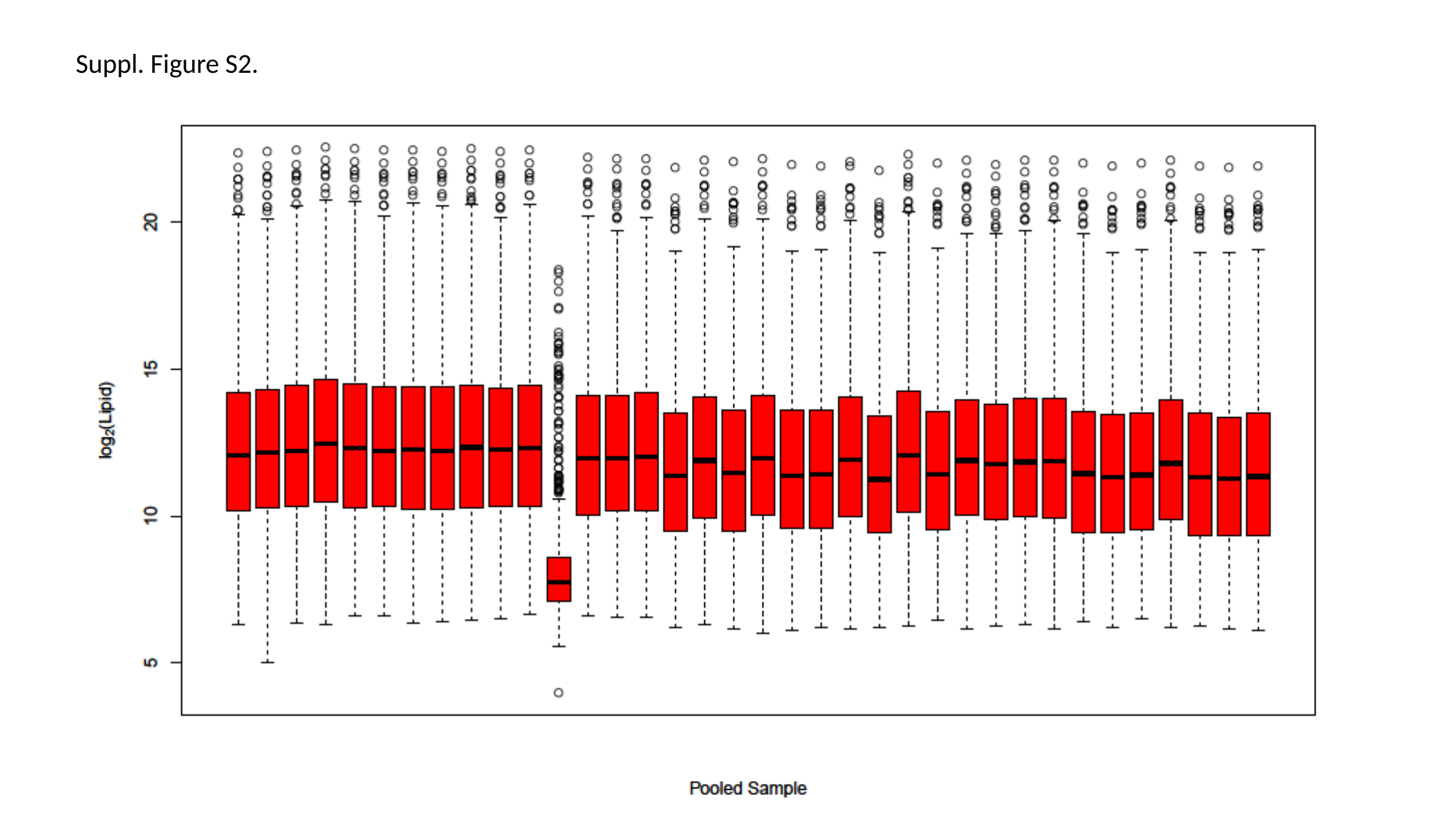

Suppl. Figure S2.

Supplement: Supplementary file 1 — Figure S1. Boxplots of the distribution of Metabolite intensity levels (on log 2 scale) for all 35 reference samples of metabolomics data. Figure S2. Boxplots of the distribution of Lipid intensity levels (on log 2 scale) for all 36 reference samples of lipidomics data. (PPTX 103 kb) [file 12882_2019_1249_MOESM1_ESM.pptx]
